# An Alternative Non-Conduit Repair Strategy for Tetralogy of Fallot With Short Segment Pulmonary Atresia

**DOI:** 10.7759/cureus.63241

**Published:** 2024-06-26

**Authors:** Anupam Das, Rengarajan Rajagopal, Palleti Rajashekar

**Affiliations:** 1 Cardiothoracic Vascular Surgery, All India Institute of Medical Sciences, Jodhpur, Jodhpur, IND; 2 Radiodiagnosis, All India Institute of Medical Sciences, Jodhpur, Jodhpur, IND; 3 Cardiothoracic Vascular Surgery, All India Institute of Medical Sciences, New Delhi, New Delhi, IND

**Keywords:** homograft, autologous pericardium, conduit salvage, hypoplastic pulmonary atresia, tetralogy of fallot

## Abstract

Tetralogy of Fallot (TOF) with pulmonary atresia is a subset in which it becomes imperative to use an artificial conduit in most cases. The atresia of the pulmonary artery can occur at various levels and be of variable lengths. For long segment pulmonary atresia, a right ventricle to pulmonary artery conduit is unavoidable in patients otherwise suitable for complete bi-ventricular repair with no major aortopulmonary collaterals, based on McGoon and Nakata indices. However, for patients with membranous pulmonary atresia and short segment atresia of the main pulmonary artery, we describe an alternative technique that avoids the use of handmade conduits or bovine jugular vein grafts and utilizes the concept of a monocusp with restoration of continuity from the right ventricular infundibulum to the distal main pulmonary artery. A seven-year-old girl diagnosed with TOF and pulmonary atresia underwent a right ventriculotomy with ventricular septal defect closure. The narrowed outflow tract was widened, and an anastomosis was made directly between the right ventricle and the pulmonary artery. A monocusp was fashioned from autologous pericardium, and the pulmonary artery was reconstructed using bovine pericardium. In TOF with pulmonary atresia, conventional surgery typically uses a valved conduit to connect the right ventricular outflow tract (RVOT) to the pulmonary artery. However, in cases like ours, direct anastomosis is possible due to a long confluent pulmonary segment. This alternative technique eliminates the need for an artificial conduit and may prevent associated problems. It also allows for potential growth of the neo-annulus and pulmonary segment. The risk of reoperation remains due to possible monocusp failure.

## Introduction

Tetralogy of Fallot (TOF) with pulmonary atresia, in most cases, mandates the use of an artificial right ventricle to pulmonary artery conduit. The use of a conduit carries future implications of conduit stenosis, failure of future growth, and the need for reoperations for conduit modifications [1]. For patients with long-segment pulmonary atresia, either a bovine jugular vein graft or a hand-sewn conduit can be implanted [2]. For short-segment pulmonary atresia or membranous pulmonary atresia, we describe our direct anastomosis technique, which avoids the use of an artificial conduit and allows for the future growth of the newly formed annulus and the pulmonary artery.

## Case presentation

**Figure 1:**
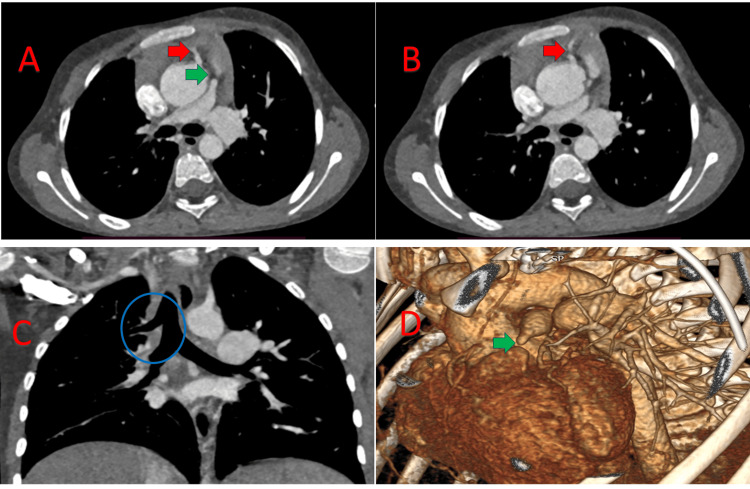
CT image showing the large RV branch from the RCA crossing the RVOT (red arrow) and the short segment pulmonary atresia (green arrow) (A); large RV branch (red arrow) (B); bronchus suis/tracheal bronchus, a rare anatomical variant where an accessory bronchus originates directly from the supracarinal trachea, known as pig bronchus when the entire upper lobe (right side) is supplied by this bronchus, as in this case (C); 3D reconstructed image showing short segment pulmonary atresia (green arrow) (D). RV: Right Ventricle; RCA: Right Coronary Artery; RVOT: Right Ventricular Outflow Tract.

Institutional Review Board waived approval for the publication of the surgical technique, and written informed consent was obtained from the patient’s parents. A seven-year-old girl (body weight 19.2 kilograms) presented with failure to thrive, central cyanosis from six months of age, and easy fatigability and shortness of breath from six years of age. Detailed evaluation revealed central cyanosis, clubbing, failure to thrive (BMI 15.8 kg/m^2^), normal position of cardiac apical impulse, single S2 (no P2), silent precordium, and a room air SpO2 of 68%. 2D transthoracic echocardiography diagnosed TOF with a large mal-aligned non-restrictive subaortic ventricular septal defect, aortic override, severe infundibular stenosis, pulmonary atresia with confluent branch pulmonary arteries (PAs), and a left-sided aortic arch. CT angiography revealed TOF with short-segment pulmonary atresia, confluent branch PAs (distance between right ventricular outflow tract (RVOT) and patent MPA 5mm), significant ostio-proximal stenosis of the left pulmonary artery (LPA) measuring 4.5mm at the ostium, 20.2mm at the hilum, right pulmonary artery (RPA) 12.5mm at the ostium, 10.5mm at the hilum with a patent ductus arteriosus (PDA) measuring 5.5mm (McGoon ratio 2.5, Nakata index 161mm^2^/m^2^), and multiple aorto-pulmonary collaterals with clockwise rotation of the aortic root and a large right ventricular branch from the right coronary artery coursing in the anterior right ventricular wall, along with bronchus suis (Figures 1A, 1B, 1D).

Two major aortopulmonary collaterals were noted: one arising from the descending thoracic aorta measuring 3mm in size at the level of the second thoracic vertebra, having a tortuous course draining into the left pulmonary artery just as it entered the hilum, and a second collateral measuring 4mm arising from the first part of the left subclavian artery, draining into the left pulmonary artery at the level of the hilum of the left lung. Neither was found to be the sole supply of any lung segment.

**Figure 2:**
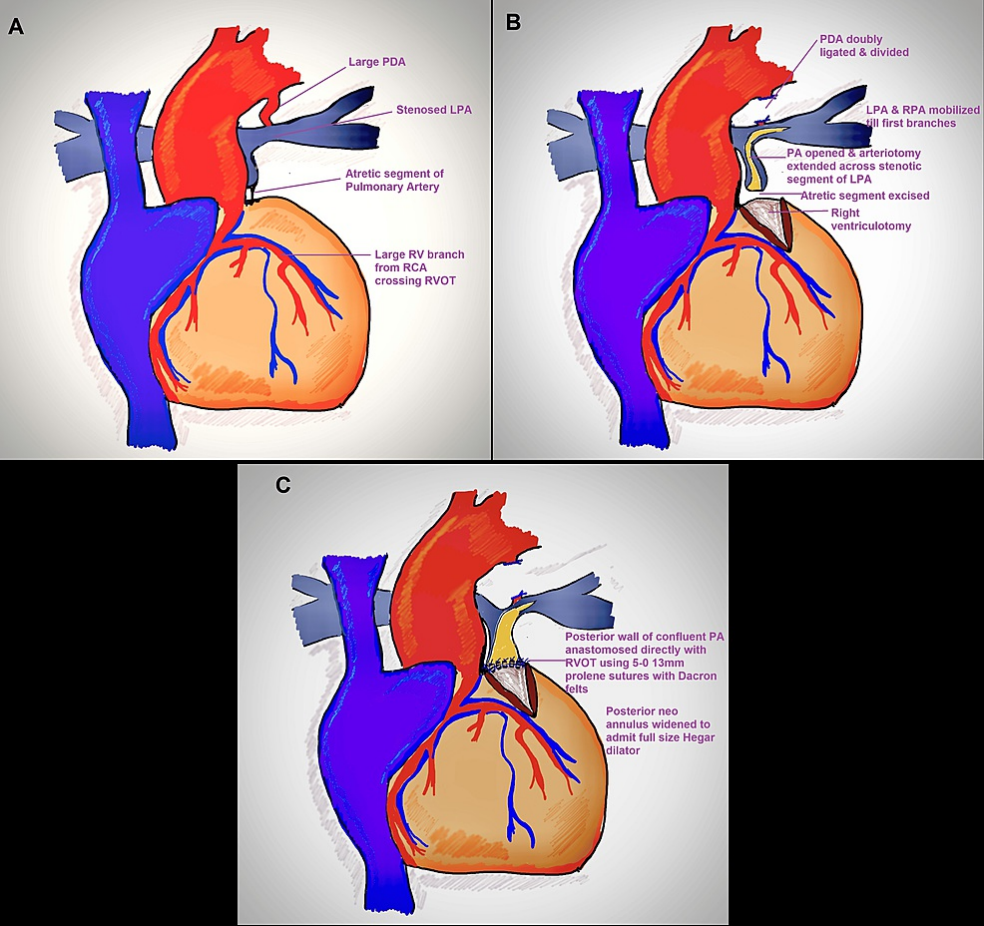
Intraoperative anatomy: Pulmonary atresia with large PDA and confluent branch PAs, showing proximal LPA stenosis (A); PDA divided, and LPA and RPA mobilized, with pulmonary arteriotomy and right ventriculotomy performed separately (B); direct anastomosis of the distal confluent pulmonary artery segment to the RVOT using 5-0 13mm pledgeted prolene sutures (C). PDA: Patent Ductus Arteriosus; PA: Pulmonary Artery; LPA: Left Pulmonary Artery; RPA: Right Pulmonary Artery; RVOT: Right Ventricular Outflow Tract.

The repair was undertaken with standard aorto-bicaval cannulation under moderate hypothermia (28°C). After a median sternotomy, the pericardium was marsupialized vertically and to the right, and reflected onto the left. Intraoperative evaluation revealed situs solitus, levocardia, a small MPA with a proximal 4-5mm atretic segment including at the valvular level, a small PDA, normal tricuspid valve, large 2x2cm subaortic VSD, severe infundibular valvular stenosis, large right ventricle (RV) branch crossing proximal RVOT, small ostium secundum atrial septal defect (OS-ASD), branch PAs confluent, RPA of adequate size, proximal LPA stenosis for a distance of 5-6mm with post stenotic dilatation (Figure 2A).

**Figure 3:**
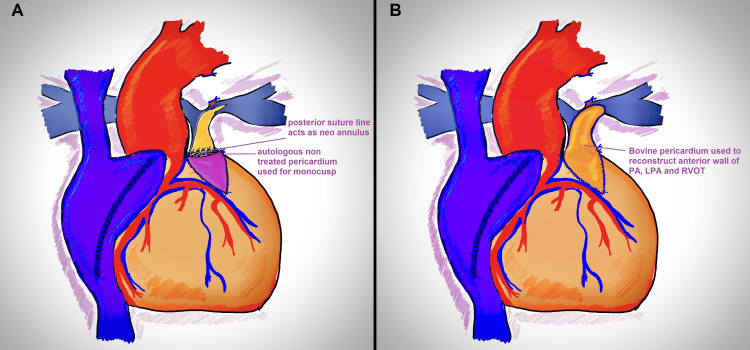
Monocusp fashioned from autologous, untreated pericardium (A); anterior pulmonary artery and right ventricular outflow tract reconstructed with bovine pericardium (B).

PDA was dissected and looped. After the initiation of cardiopulmonary bypass (CPB), the PDA was doubly ligated and divided. The LPA and RPA were dissected until their first branches (Figure 2B). The MPA was dissected free from the aorta and looped separately. 6-0 polypropylene stays were placed on the MPA and RVOT at the site of the planned ventriculotomy, distal to the large RCA branch on the RV wall. The LAD and proximal aorta acted as guiding points in marking the site of right ventriculotomy. After cardioplegia (Del Nido solution: 20 ml/kg for the first dose followed by 10 ml/kg of lignocaine-free solution at 90 minutes), right atriotomy was performed, and the VSD was closed using a Dacron patch with interrupted pledgeted 5-0 13mm polypropylene sutures in a standardized fashion. Ventriculotomy was performed between the stays (Figure 2B) and infundibular resection was done until the infundibulum admitted a 17mm Hegar dilator (full size 15mm). The MPA was opened, and the arteriotomy was extended across the stenotic segment of the LPA between the stays, a technique previously described by Roger Mee (Figure 2B). The posterior wall of the confluent pulmonary segment was directly anastomosed to the opened RVOT using interrupted 5-0 13mm Dacron pledgeted polypropylene sutures (Figure 2C). Care was taken to avoid any gaps in the suture line to prevent bleeding, as this would become virtually inaccessible after complete reconstruction. This posterior suture line was widened to approximate the diameter of the full-size Hegar dilator (15mm). Autologous pericardium was harvested and fashioned into a monocusp, and sutured to the edges of the ventriculotomy using 6-0 13mm polypropylene sutures (Figure 3A). The anterior wall of the RVOT, confluent PA segment, and LPA were reconstructed with bovine pericardium (St. Jude Medical Pericardial Patch with EnCap AC technology), ensuring both the LPA and RPA admitted a half-size Hegar dilator (11mm, Figure 3B).

It is important to be cautious about the fact that in cases of membranous or short-segment pulmonary atresia, it is possible to enter the left ventricle at the annular level if an attempt is made to open the PA and RVOT in continuity. For this technique to be successful, it is critical to mobilize the LPA and RPA until their first branches to avoid any undue kinking at the confluence. After weaning off cardiopulmonary bypass, the pRV/LV was 0.55. TEE revealed no residual shunt, no aortic or pulmonary regurgitation, and a peak RV-PA gradient of 40mmHg; the right ventricular systolic pressure, as predicted by tricuspid regurgitation, was 40 mm Hg. The duration of CPB was 134 minutes, and that of the aortic cross clamp was 105 minutes. The child was shifted with good hemodynamics on 0.1µg/kg/minute of adrenaline, 0.5µg/kg/minute of milrinone, and 0.05µg/kg/minute of noradrenaline. She was extubated on day 1, and inotropes were gradually tapered off by day 3. She was discharged on day 14 and is under regular follow-up.

**Figure 4:**
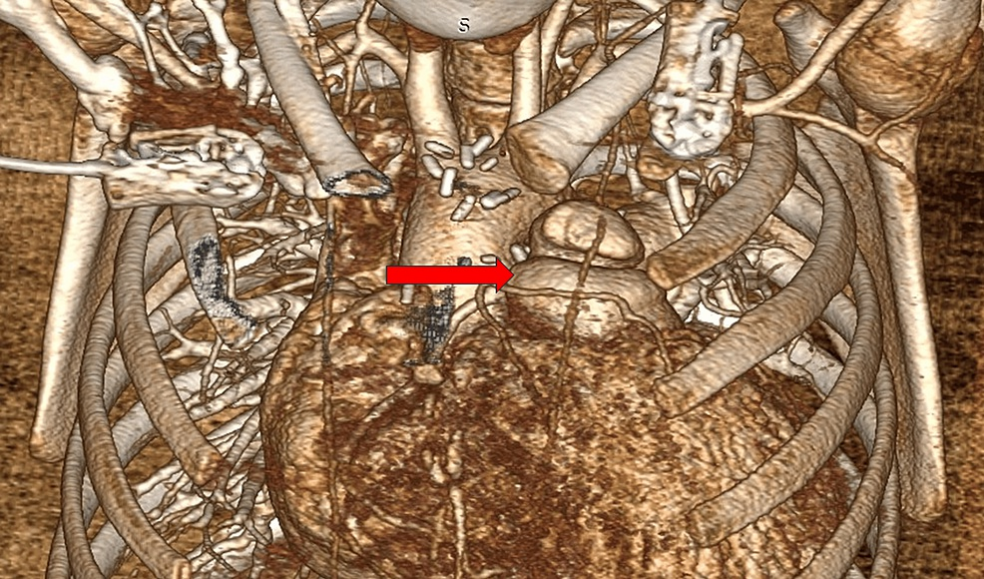
Follow-up CT showing a well-reconstructed, good-sized main pulmonary artery (red arrow).

At the 6-month follow-up, she showed good weight gain and was asymptomatic on oral enalapril (0.2mg/kg/day in two divided doses), oral digoxin (5µg/kg twice daily for 6 days a week), and oral furosemide (1mg/kg/day in two divided doses). The transthoracic echocardiography at the 6-month follow-up showed no residual VSD, no pulmonary regurgitation (well-functioning monocusp valve), a peak right ventricular outflow tract gradient of 28mmHg, and trivial tricuspid regurgitation with good biventricular function. A CT angiography (Figure 4) of the cardiac anatomy was performed, revealing a well-sized main pulmonary artery (Z-score: -0.78 for BSA) with well-sized branch pulmonary arteries and no residual stenosis. The right ventricular outflow tract was found to be widely open with no major muscle bundle causing any significant stenosis.

## Discussion

Conventionally, surgical repair for TOF with pulmonary atresia involves the use of a valved conduit to connect the RVOT to the pulmonary artery segment. Direct anastomosis, as in our case, is made feasible by the presence of a long confluent pulmonary segment and close proximity to the right ventricular outflow tract in this subset of TOF with pulmonary atresia. This alternative surgical reconstruction dispenses with the need for an artificial conduit and arguably would prevent problems inherent to the use of a conduit, and may allow growth of the neo-annulus and the pulmonary segment. The technique may not completely reduce the risk of reoperation as failure of the monocusp is possibly inherent to this technique. Jang DH et al. [1] in their analysis of 141 infants who underwent surgery for biventricular repair using an RV-PA conduit, reported conduit dysfunction (peak velocity > 3.5m/s or moderate or severe regurgitation) in 68 patients and conduit reintervention in 61 patients within five years. These patients would arguably require multiple surgeries for conduit correction or replacements. This can be prevented by the direct anastomosis technique (as in our case), which allows for growth of the main pulmonary artery segment and the neo-annulus. Pulmonary regurgitation due to the failure of the monocusp is anticipated in our technique; however, with the growth of the annulus and right ventricular outflow tract, pulmonary valve replacement always remains as a future option within the native RVOT-PA junction, which may not be the case in a repair utilizing any artificial conduit, thus reducing the number of reoperations in the future. Currently, the ideal conduit for establishing RV to PA continuity in a patient with pulmonary atresia remains unclear. Hao S et al. [2], in their analysis of midterm outcomes of bovine jugular valved conduits for RVOT reconstruction in pediatric patients, also reported conduit dysfunction in 33% of the patients within a median follow-up period of 43.5 months and conduit failure in 9.6% of patients, of whom 6 required reoperations.

In a study of 366 patients by Isomatsu Y et al. [3], who underwent establishment of RV-PA continuity using either hand-made valved equine pericardial conduits, autologous pericardial conduits, or direct anastomosis without a conduit, it was found that direct anastomosis was associated with significantly better freedom from late conduit replacement or late death than equine pericardial conduits or autologous pericardial conduits. This finding substantially adds to the evidence in favor of a non-conduit repair strategy for pulmonary atresia in selected patients.

Gupta SK et al. [4] reported a similar technique in a patient with truncus arteriosus, wherein a vascular channel arose anteriorly from the anterior aspect of the right sinus of the common trunk and, after a long course, divided into right and left pulmonary arteries. They reconstructed the posterior annulus in a similar fashion, and the anterior wall was fashioned using a monocusp pulmonary homograft. Reconstruction of the MPA has also been achieved using the left atrial appendage and a monocusp valve in patients with truncus arteriosus [5] and TOF with pulmonary atresia, either for complete repair [3] or as a neonatal palliation to establish the right ventricular to pulmonary artery connection [6]. These studies have reported good and acceptable results with this direct anastomosis technique; however, it may carry certain concerns. Namely, the use of autologous pericardium for RVOT-PA reconstruction carries the risk of aneurysmal dilatation, and the use of heterologous pericardium or glutaraldehyde-treated autologous pericardium may result in calcification and also limits the future growth potential [7]. Additionally, it may require greater expertise (with regard to monocusp fashioning, avoiding kinking of branch PAs) and longer durations of CPB than what may be required for conduit repair.

## Conclusions

The direct anastomosis technique holds significant sway in a carefully chosen cohort of patients, most notably among younger patients, where recurrent surgical interventions for conduit substitutions can be circumvented, thus underscoring the paramount importance of fostering potential growth within the neo-pulmonary annulus. The article delineates a meticulous and methodical approach toward realizing the desired outcome. In such instances, while the creation of a monocusp is not deemed obligatory, it stands as a judicious measure to preempt early free pulmonary regurgitation, thus averting the consequential burden of volume overload upon an already hypertrophied right ventricle. Such prophylaxis is paramount, as it mitigates the likelihood of a prolonged cardiac surgical ICU stay and heightened inotropic support.
